# Supplementary material for: Lineage-informative microhaplotypes for recurrence classification and spatio-temporal surveillance of Plasmodium vivax malaria parasites
Source: Nat Commun. 2024 Aug 8;15:6757. doi: 10.1038/s41467-024-51015-3 (PMC11310204; doi:10.1038/s41467-024-51015-3)
Supplement: Supplementary file 1 — Supplementary Information [file 41467_2024_51015_MOESM1_ESM.pdf]

Supplementary Information

Lineage-informative microhaplotypes for recurrence classification and spatio-temporal surveillance of *Plasmodium vivax* malaria parasites

Sasha V. Siegel[1,2], Hidayat Trimarsanto[2,3], Roberto Amato[1], Kathryn Murie[1], Aimee R. Taylor[4], Edwin Sutanto[5], Mariana Kleinecke[2], Georgia Whitton[1], James A. Watson[6,7], Mallika Imwong[8], Ashenafi Assefa[9], Awab Ghulam Rahim[10,11], Nguyen Hoang Chau[7], Tran Tinh Hien[7], Justin A. Green[12], Gavin C.K.W. Koh[13], Nicholas J. White[6,10], Nicholas Day[6,10], Dominic P. Kwiatkowski[1], Julian C. Rayner[14], Ric N. Price[2,6,10], Sarah Auburn[2,6]

[1] Wellcome Sanger Institute, Hinxton, Cambridge CB10 1SA, UK
[2] Menzies School of Health Research and Charles Darwin University, Darwin, Northern Territory 0811, Australia
[3] Eijkman Institute for Molecular Biology, National Research and Innovation Agency, Jakarta 10430, Indonesia
[4] Institut Pasteur, University de Paris, Infectious Disease Epidemiology and Analytics Unit, Paris, France
[5] Exeins Health Initiative, Jakarta Selatan 12870, Indonesia
[6] Centre for Tropical Medicine and Global Health, Nuffield Department of Medicine, University of Oxford, OX3 7LJ, UK
[7] Oxford University Clinical Research Unit, Hospital for Tropical Diseases, 764 Vo Van Kiet, W.1, Dist.5, Ho Chi Minh City, Vietnam
[8]Department of Molecular Tropical Medicine and Genetics, Faculty of Tropical Medicine, Mahidol University, Bangkok, Thailand
[9] Ethiopian Public Health Institute, Addis Ababa, Ethiopia
[10] Mahidol-Oxford Tropical Medicine Research Unit, Faculty of Tropical Medicine, Mahidol University, Bangkok 10400, Thailand
[11] Afghan International Islamic University, Kabul, Afghanistan
[12] Formerly GlaxoSmithKline, Brentford, UK
[13] Department of Infectious Diseases, Northwick Park Hospital, Harrow, UK
[14] Cambridge Institute for Medical Research, University of Cambridge, Hills Road, Cambridge, CB2 0XY, UK

Deceased: Dominic P. Kwiatkowski

Corresponding author: Dr Sarah Auburn, Sarah.Auburn@Menzies.edu.au, Menzies School of Health Research, PO Box 41096, Casuarina, Darwin, NT 0811, Australia; Tel: (+61) 8 8946 8503

Contents

Supplementary Figure 1. *P. vivax* incidence map illustrating regional country groupings. **3**

Supplementary Figure 2. Comparative accuracy in relatedness prediction for Random and High-diversity microhaplotype panels, and the 38-SNP Broad barcode. **4**

Supplementary Figure 3. Correlations between microhaplotype and genomic estimates of IBD in Pv4 dataset by region. **6**

Supplementary Table 1. Regional patterns of within-host infection diversity. **7**

Supplementary Figure 4. Regional microhaplotype-based infection networks.................................... **9**

Supplementary Table 2. Summary of median MCC scores from comparative evaluations of country prediction performance between the SNP panels.. **10**

Supplementary Figure 5. Clustering patterns of the independent validation *P. vivax* dataset relative to MalariaGEN Pv4. .... **12**

Supplementary Figure 6. Correlations between microhaplotype and genomic estimates of IBD in external, independent validation dataset by region. **14**

Supplementary Figure 7. Comparative accuracy in relatedness prediction at high diversity microhaplotype panels of different marker sizes (50, 100, 150, 200, 250). **14**

Supplementary Note 1. Detailed methods on the *P. vivax* independent validation dataset **15**

References **17**

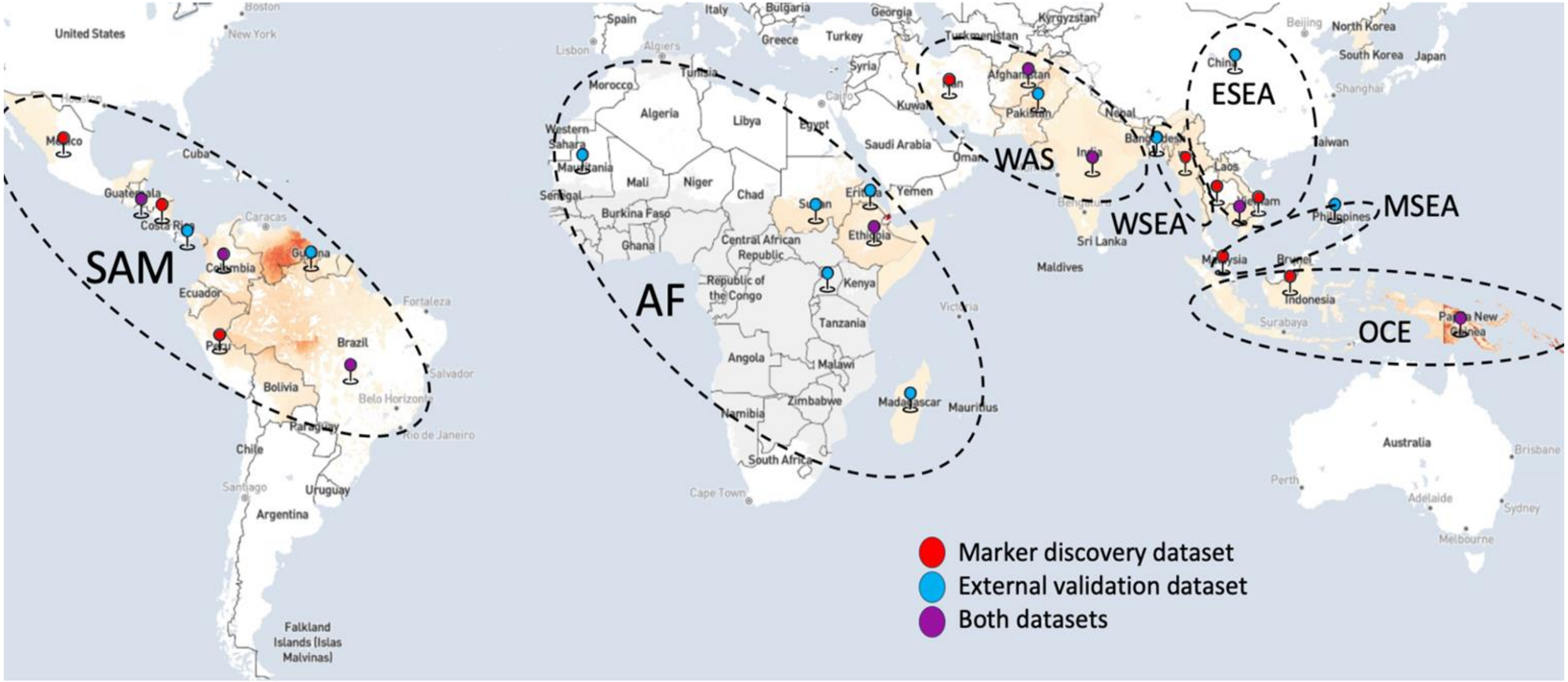

Supplementary Figure 1. *P. vivax* incidence map illustrating regional country groupings.

The baseline *P. vivax* incidence map was derived from the Malaria Atlas Project (MAP) and presents the number of newly diagnosed *P. vivax* cases per 1,000 population in 2020[1]. The labelled, dashed black lines indicate the boundaries of the geographic regions included in the analyses: SAM (South America), AF (Africa), WAS (West Asia), WSEA (West Southeast Asia), ESEA (East Southeast Asia), MSEA (Maritime Southeast Asia) and OCE (Oceania). Pins indicate the countries included in each regional grouping, with red pins indicating countries that were represented with samples in the marker discovery dataset (from Pv4), blue pins for those that were part of the external validation dataset (non-Pv4), and purple pins for countries that were present in both datasets. Most of Africa does not have endemic *P. vivax* due to widespread duffy negativity and is generally geographically restricted to the Horn of Africa. The data represented in this study captures the majority of countries where *P. vivax* is prevalent.

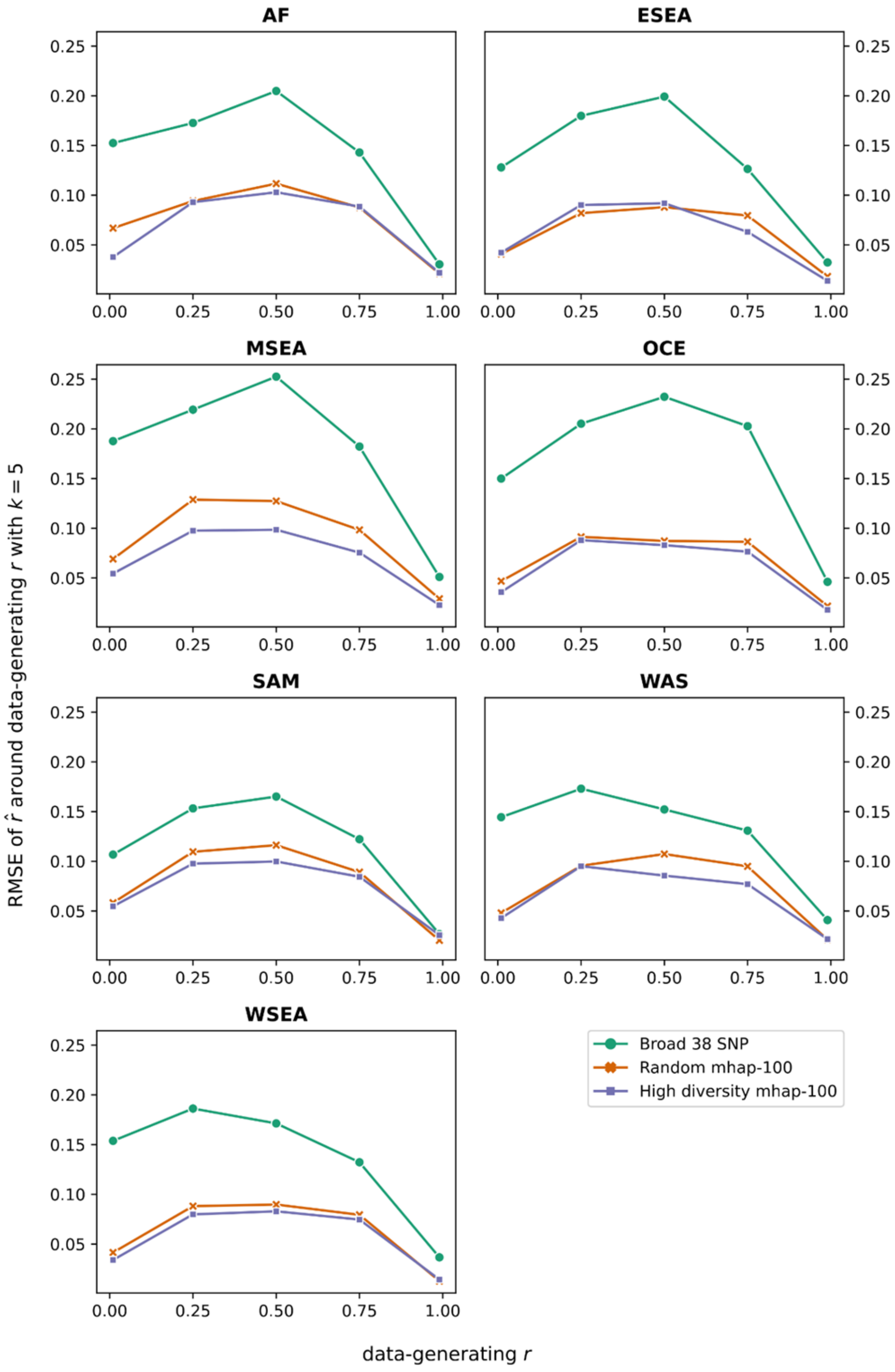

Supplementary Figure 2. Comparative accuracy in relatedness prediction for Random and High-diversity microhaplotype panels, and the 38-SNP Broad barcode.

Root mean square error (RMSE) of relatedness estimates based on data simulated using various data-generating relatedness and switch rate parameters, r and k, respectively. The simulations were informed by data on n=615 independent *P. vivax* samples. Data are presented on 3 marker panels: High-diversity SNP microhaplotype panel, Random-SNP microhaplotype panel and 38 Broad barcode biallelic SNPs. Panel comparisons are presented by geographic region; AF (Africa), ESEA (East Southeast Asia), MSEA (Maritime Southeast Asia), OCE (Oceania), SAM (South America), WAS (West Asia) and WSEA (West Southeast Asia).

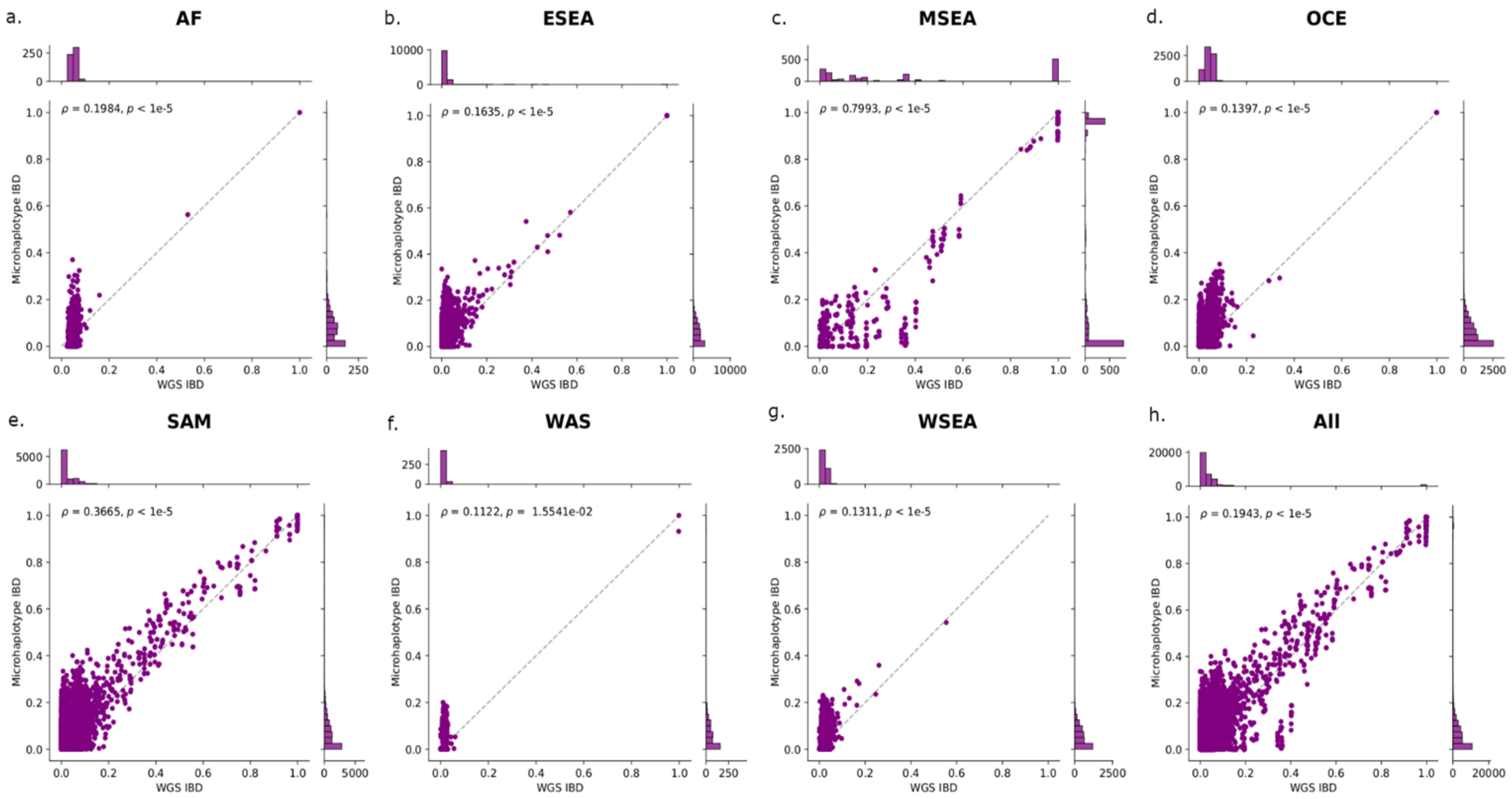
a.
AF
ρ = 0.1984, p < 1e-5
b.
ESEA
ρ = 0.1635, p < 1e-5
c.
MSEA
ρ = 0.7993, p < 1e-5
d.
OCE
ρ = 0.1397, p < 1e-5
e.
SAM
ρ = 0.3665, p < 1e-5
f.
WAS
ρ = 0.1122, p = 1.5541e-02
g.
WSEA
ρ = 0.1311, p < 1e-5
h.
All
ρ = 0.1943, p < 1e-5
Microhaplotype IBD
WGS IBD

Supplementary Figure 3. Correlations between microhaplotype and genomic estimates of IBD in Pv4 dataset by region.

Panels a) to g) represent Africa (AF), East Southeast Asia (ESEA), Maritime Southeast Asia (MSEA), Oceania (OCE), South America (SAM), West Asia (WAS), and West Southeast Asia (WSEA). The microhaplotype and whole genome sequence (WGS) IBD estimates reflect pairwise estimates at the High-diversity SNP microhaplotype panel and a set of 898,448 genome-wide SNPs. All calculations were performed using *hmmIBD* on the 615 monoclonal sample set. Correlations were assessed with Spearman's rho statistic (using a paired test) and presented with the associated p-value. At an alpha of 0.05, significantly positive correlations were observed in all regions.

Region	% Isolates *F*ws <0.95	% Isolates COI >1	% Isolates Fws<0.95 vs COI>1 (X^2, *p*-value*)	Median *F*ws (Min-max): all isolates	Median *F*ws (Min-max): isolates COI=1	Median *F*ws (Min-max): isolates COI>1	*F*ws in COI=1 vs COI>1 (W, *p*-value**)
AF	27.7 (13/47)	23.4 (11/47)	X2 = 0.06, p = 0.813	0.995 (0.303-0.967)	0.996 (0.888-0.999)	0.669 (0.303-0.919)	W = 394, p = 2.30e-10
ESEA	42.4 (111/262)	38.9 (102/262)	X2 = 0.51, p = 0.477	0.992 (0.215-1.000)	0.996 (0.850-1.000)	0.649 (0.215-0.926)	W = 16307, p < 2.2e-16
MSEA	22.4 (17/76)	17.1 (13/76)	X2 = 0.374, p = 0.541	0.994 (0.516-1.000)	0.995 (0.891-1.000)	0.827 (0.516-0.921)	W = 816, p = 1.07e-08
OCE	41.5 (85/205)	36.1 (74/205)	X2 = 0.800, p = 0.371	0.993 (0.193-1.000)	0.996 (0.867-1.000)	0.710 (0.193-0.915)	W = 9656, p < 2.2e-16
SAM	15.1 (24/159)	13.2 (21/159)	X2 = 0.104, p = 0.748	0.997 (0.457-1.000)	0.997 (0.743-1.000)	0.794 (0.457-0.999)	W = 2760, p = 1.31e-11
WAS	32.6 (15/46)	28.3 (13/46)	X2 = 0.051, p = 0.821	0.996 (0.434-1.000)	0.996 (0.873-1.000)	0.781 (0.434-0.815)	W = 429, p = 9.83e-12
WSEA	33.1 (42/127)	30.7 (39/127)	X2 = 0.073, p = 0.788	0.996 (0.225-1.000)	0.997 (0.907-1.000)	0.648 (0.225-0.915)	W = 3431, p < 2.2e-16

Supplementary Table 1. Regional patterns of within-host infection diversity.

Measures were conducted on n=922 high-quality biologically independent samples from Pv4. *Pearson's Chi-squared test with Yates' continuity correction. **1-sided Mann-Whitney U testing the hypothesis that the *F*ws in the COI=1 infection group is not larger than in the COI>1 infection group.

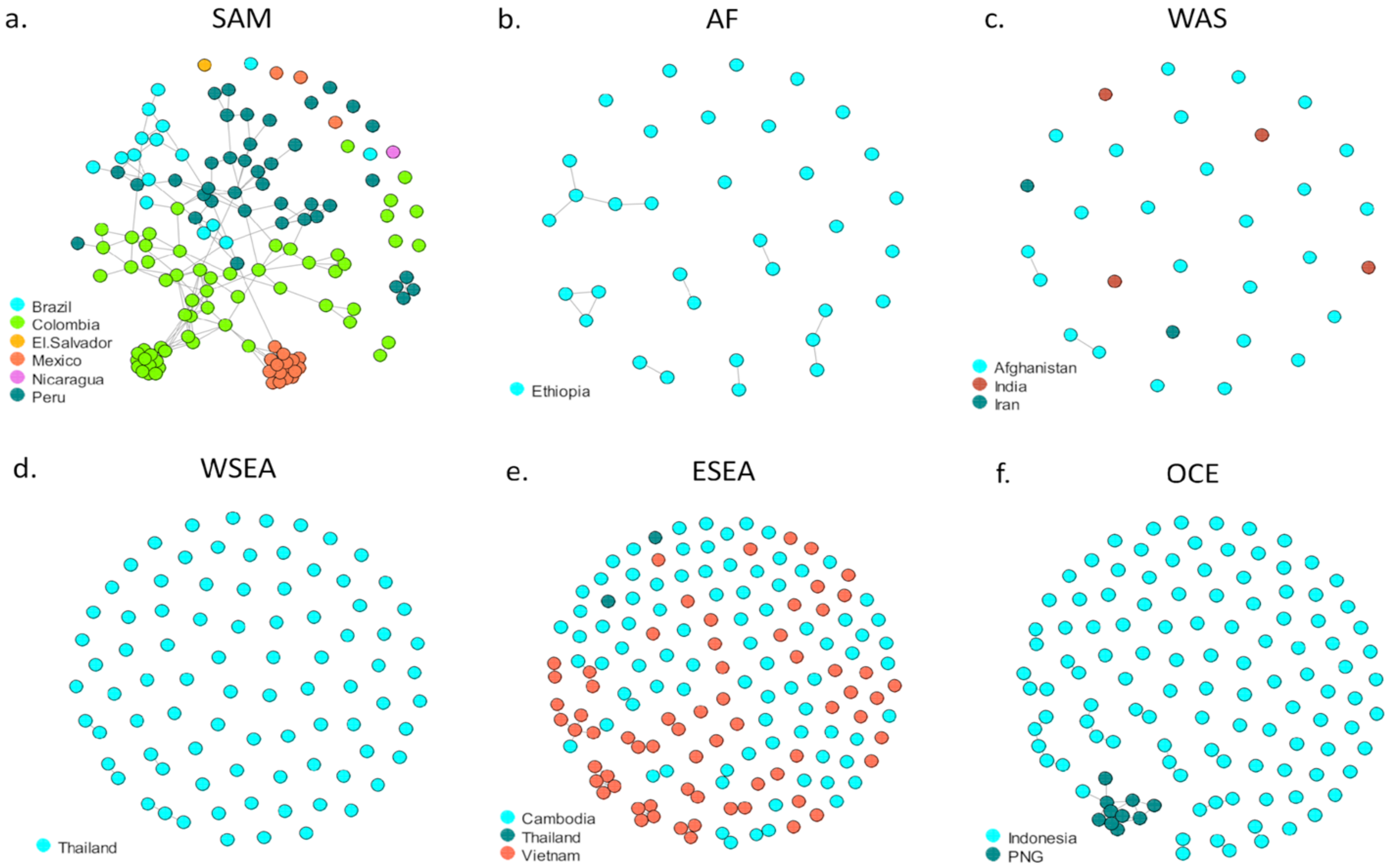
a.
SAM
Brazil
Colombia
El.Salvador
Mexico
Nicaragua
Peru
b.
AF
Ethiopia
c.
WAS
Afghanistan
India
Iran
d.
WSEA
Thailand
e.
ESEA
Cambodia
Thailand
Vietnam
f.
OCE
Indonesia
PNG

Supplementary Figure 4. Regional microhaplotype-based infection networks.

Panels a) to e) represent South America (SAM), Africa (AF), West Asia (WAS), West Southeast Asia (WSEA), East Southeast Asia (ESEA) and Oceania (OCE). The networks were generated from the High-diversity SNP microhaplotype panel in the 615 monoclonal sample set. The isolates from Maritime Southeast Asia (MSEA) were mostly from Malaysia (57/59) and are represented. Each circle reflects an infection, colour-coded by country, and line lengths reflect relatedness (shorter lines reflect greater relatedness) at a connectivity threshold of minimum identity by descent (IBD) 0.25 (half-siblings or greater relatedness). Where two circles are not connected by a line, the estimated IBD between those infections was below the given threshold of 0.25 (i.e. they were not inferred to be highly related). Alternative IBD thresholds can be applied to detect more distant connectivity between infections. The distance between infections (circles) that are not connected by lines does not reflect the relatedness between those infections. The largest infection networks were observed in South America. Small networks were observed in other regions, but most infections were unrelated at a minimal IBD threshold of 0.25 (consistent with half-siblings or closer). In East Southeast Asia, cross-border networks were observed between Cambodia, Thailand and Vietnam.

Country	BR38	GEO33	GEO50	GEO55	MHAP-3_10
Afghanistan	0.835	0.850	0.908	0.889	0.946
Bangladesh	0.784	1.000	1.000	1.000	1.000
Bhutan	1.000	1.000	1.000	1.000	1.000
Brazil	0.892	0.892	1.000	1.000	1.000
Cambodia	0.520	0.756	0.823	0.874	0.866
China	0.864	1.000	1.000	1.000	1.000
Colombia	0.870	1.000	1.000	1.000	1.000
Ethiopia	0.923	0.933	1.000	1.000	1.000
India	0.794	0.864	0.892	0.864	1.000
Indonesia	0.913	0.939	0.985	1.000	0.985
Iran	1.000	1.000	0.892	1.000	1.000
Madagascar	1.000	1.000	1.000	1.000	1.000
Malaysia	0.784	0.933	0.933	0.933	0.933
Mexico	1.000	1.000	1.000	1.000	1.000
Myanmar	0.620	0.910	0.910	0.892	1.000
Papua New Guinea	0.344	0.812	1.000	1.000	1.000
Peru	0.817	0.940	1.000	1.000	1.000
Philippines	0.661	0.892	0.864	1.000	1.000
Sudan	1.000	1.000	1.000	1.000	1.000
Thailand	0.591	0.930	0.975	0.976	1.000
Vietnam	0.284	0.523	0.672	0.769	0.749
Pooled median	0.835	0.933	1.000	1.000	1.000
Pooled min	0.284	0.523	0.672	0.769	0.749
Pooled Q1	0.753	0.892	0.910	0.965	1.000

Supplementary Table 2. Summary of median MCC scores from comparative evaluations of country prediction performance between the SNP panels.

Comparisons were undertaken between the 494 SNPs in the High-diversity 100 microhaplotype panel (MHAP-3_10), the 38-SNP Broad barcode (BR38), and the 33-, 50- and 55-SNP GEO panels (GEO33, GEO50 and GEO55 respectively). The median Matthews correlation coefficient (MCC) summary statistics are based on 500 repeats of the stratified 10-fold cross-validation using the Bi-Allele Likelihood (BALK) classifier in a set of $n = 799$ biologically independent samples from 21 countries (each with n ≥4)[2].

a.

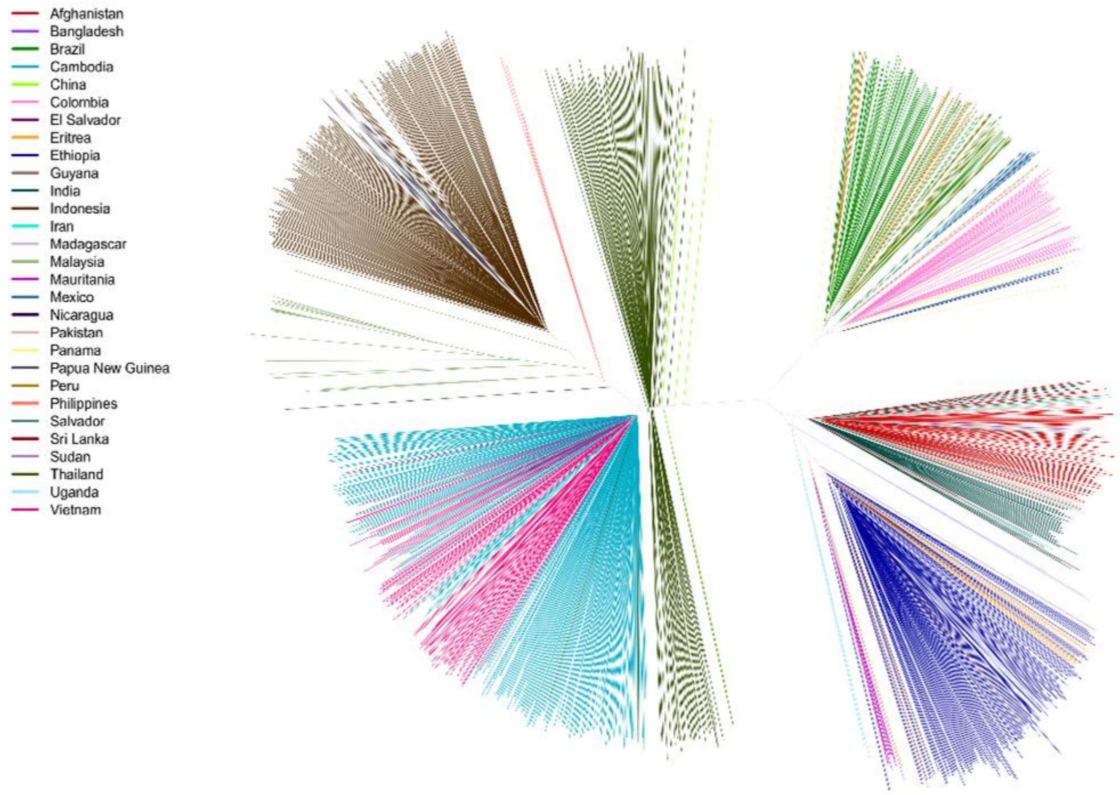
Afghanistan
Bangladesh
Brazil
Cambodia
China
Colombia
El Salvador
Eritrea
Ethiopia
Guyana
India
Indonesia
Iran
Madagascar
Malaysia
Mauritania
Mexico
Nicaragua
Pakistan
Panama
Papua New Guinea
Peru
Philippines
Salvador
Sri Lanka
Sudan
Thailand
Uganda
Vietnam

b.

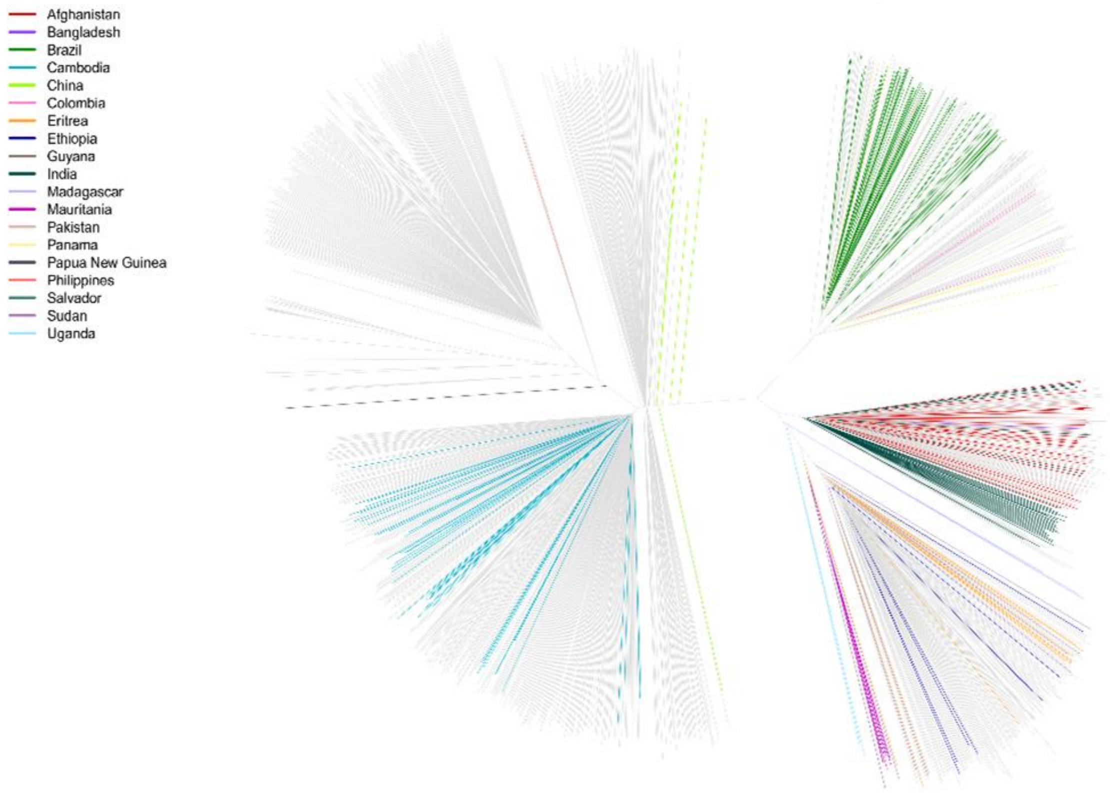
Afghanistan
Bangladesh
Brazil
Cambodia
China
Colombia
Eritrea
Ethiopia
Guyana
India
Madagascar
Mauritania
Pakistan
Panama
Papua New Guinea
Philippines
Salvador
Sudan
Uganda

c.

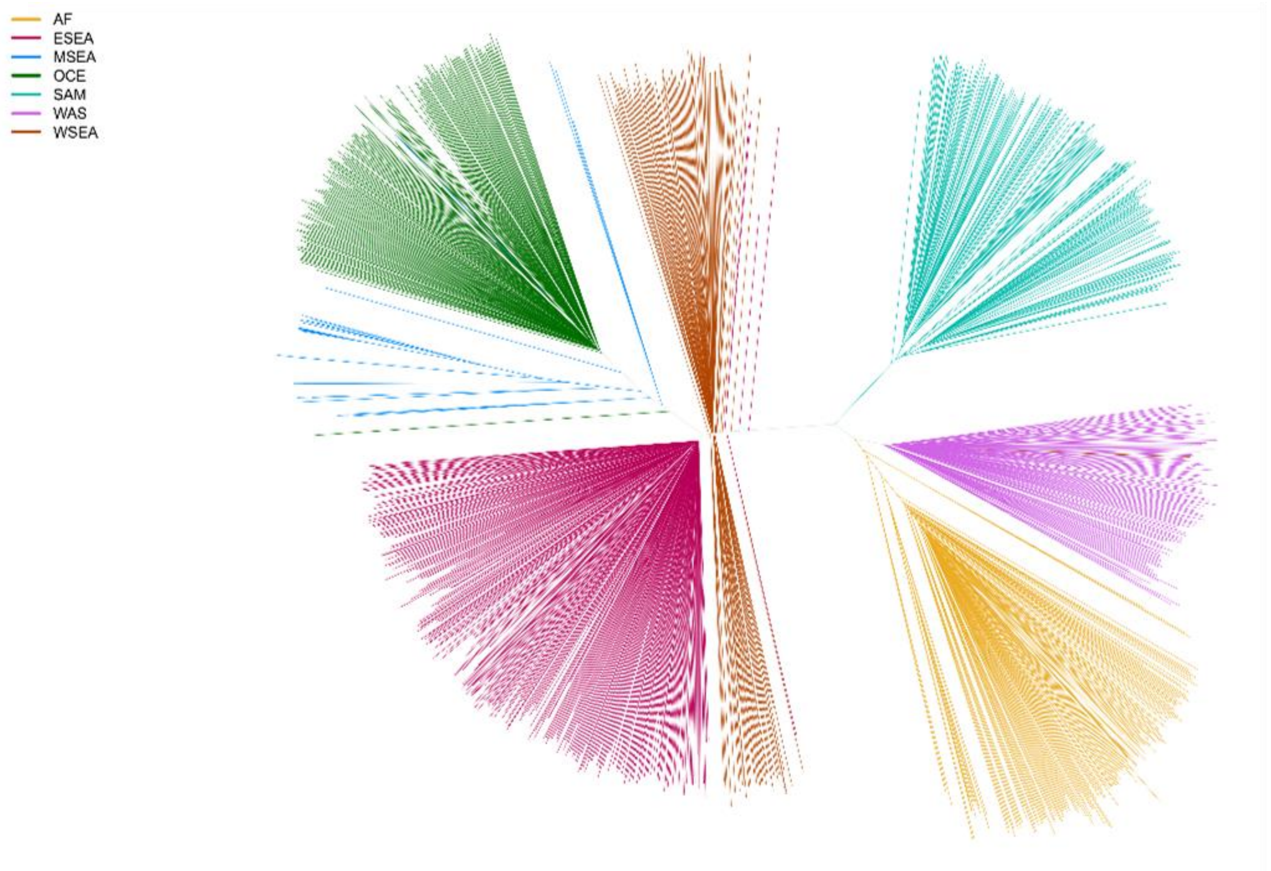

Supplementary Figure 5. Clustering patterns of the independent validation *P. vivax* dataset relative to MalariaGEN Pv4.

The panels present neighbour-joining trees with differing colouring to highlight different sample sets, regional and country origins. The tree was constructed using the n=1,031 samples annotated as 'Analysis_set' in Pv4 dataset and n=324 independent validation samples in the external (non-Pv4) dataset. Panel a) presents the tree coloured by individual countries for all samples. Panel b) presents the tree coloured by countries for n=324 samples from external (non-Pv4) dataset while Pv4 samples were greyed out. Panel c) presents the same tree coloured by regions for all samples, illustrating the regional grouping classifications; AF (Africa), ESEA (East Southeast Asia), Maritime Southeast Asia (MSEA), West Asia (WAS), West Southeast Asia (WSEA) and South America (SAM), indicating that all external (non-Pv4) dataset clustered on the same regions as the MalariaGEN Pv4 dataset.

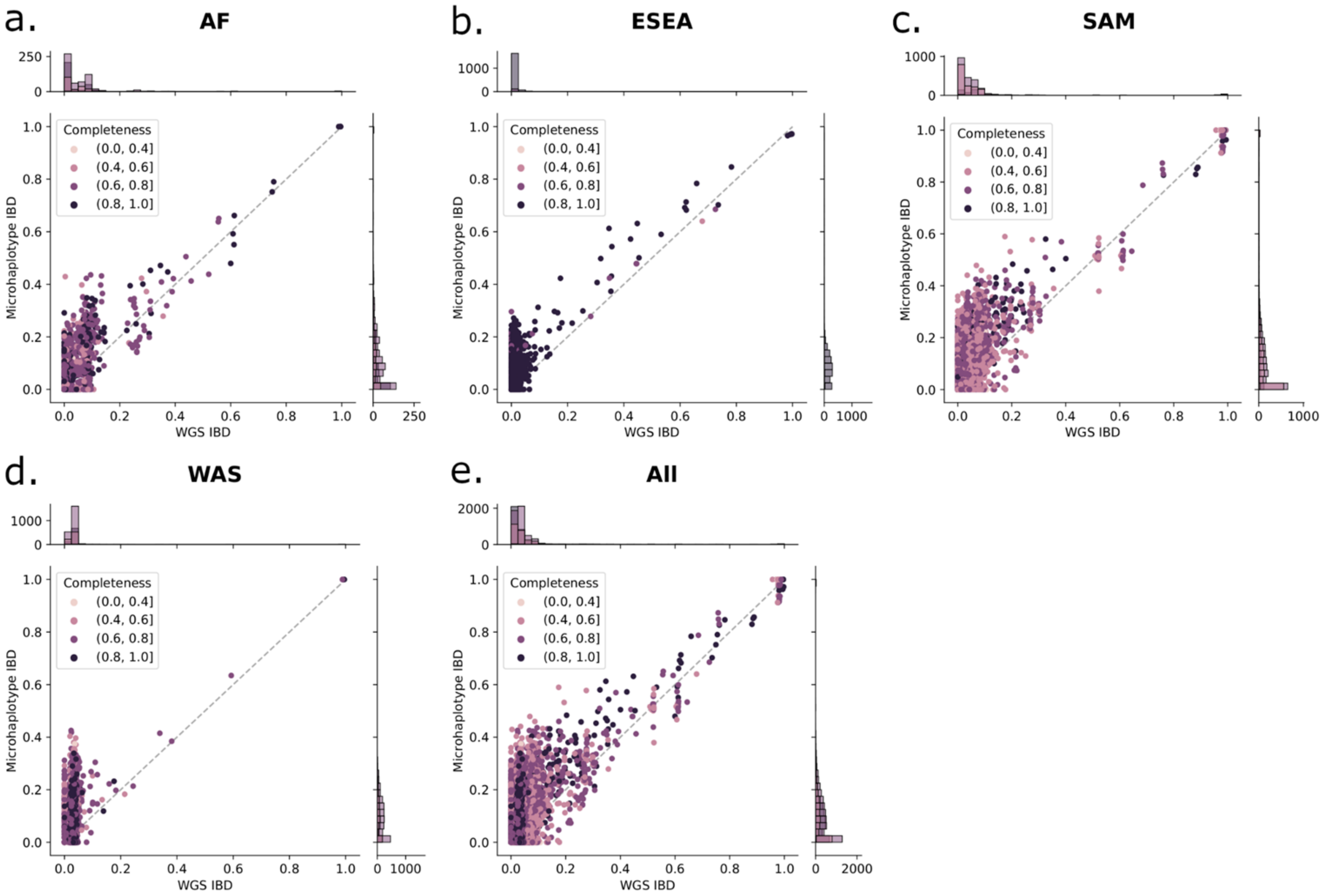
a.
AF
b.
ESEA
c.
SAM
d.
WAS
e.
All
Completeness
(0.0, 0.4]
(0.4, 0.6]
(0.6, 0.8]
(0.8, 1.0]
Microhaplotype IBD
WGS IBD

Supplementary Figure 6. Correlations between microhaplotype and genomic estimates of IBD in external, independent validation dataset by region.

Panels a) to e) represent Africa (AF), East Southeast Asia (ESEA), Western Asia (WAS), South America (SAM) and the global dataset (All). The microhaplotype and whole genome sequence (WGS) IBD estimates reflect pairwise estimates at the High-diversity SNP microhaplotype panel and genome-wide SNPs. All calculations were performed using *hmmIBD* on the 288 monoclonal sample set. Individual points are colour-coded by completeness (i.e. fraction of positions with genotype calls) ranging from light purple (up to 40% positions called) to dark purple (up to 100% positions called). Correlations were assessed with Spearman's rho statistic (using a paired test). At an alpha of 0.05, significantly positive correlations were observed in AF (rho =0.5877, $p < 1e\text{-}5$), ESEA (rho = 0.2722, $p < 1e\text{-}5$), SAM (rho = 0.5047, $p < 1e\text{-}5$), WAS (rho = 0.1249, $p < 1e\text{-}5$), and All (rho = 0.3755, $p < 1e\text{-}5$). The histograms on the axes present the respective distribution of the microhaplotype-based IBD estimates (right hand y-axis) and the WGS-based IBD estimates (upper x-axis). The microhaplotypes consistently slightly overestimate IBD relative to WGS data at relatedness values less than 0.2. This could be related to intrinsic properties of the *hmmIBD* algorithm, which was not optimally designed for use with microhaplotype data in its initial iteration. However, this apparent overestimation artifact would not be of particular concern for recurrence characterization, where the primary question is whether parasite pairs are identical (r=1), siblings (r=0.5), or even possibly half-siblings (r=0.25). This analysis highlights the need for dedicated methods and tools development for effectively using microhaplotype markers from the wider community.

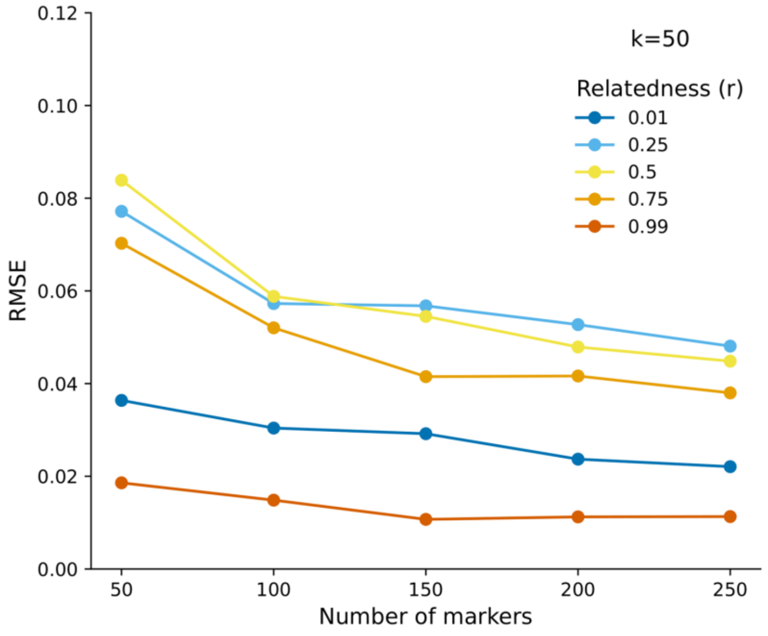

Supplementary Figure 7. Comparative accuracy in relatedness prediction at high diversity microhaplotype panels of different marker sizes (50, 100, 150, 200, 250).

Root mean square error (RMSE) of relatedness estimates based on data simulated using various data-generating relatedness parameters, *r* (range 0, 0.25, 0.5, 0.75 and 1.0) and switch rate parameter k = 50. All calculations were performed using *paneljudge* on the 615 independent, monoclonal MalariaGEN Pv4 samples.

Supplementary Note 1. Detailed methods on the *P. vivax* independent validation dataset

Data Sources

The independent validation dataset was derived from published, open-access Illumina paired-end genome wide *P. vivax* data from samples that were not represented in the MalariaGEN Pv4 dataset[3]. The FASTQ read files for the samples were downloaded from the European Nucleotide Archive (ENA) or National Center for Biotechnology Information (NCBI) Entrez Short Read Archive (SRA) database utilizing SRA-Repo (https://github.com/vivaxgen/sra-repo) using the sample SRA accession numbers. From an initial set of 836 samples, a total of 827 samples with proper Illumina paired-end data were downloaded from 8 published studies that were not compiled in Pv4 as outlined below[4-11].

***P. vivax* independent sample publication sources**
Benavente, E. D. *et al*. Distinctive genetic structure and selection patterns in *Plasmodium vivax* from South Asia and East Africa. *Nat. Commun*. 12, 3160 (2021).
Buyon, L. E. *et al*. Population genomics of *Plasmodium vivax* in Panama to assess the risk of case importation on malaria elimination. *PLoS Negl. Trop. Dis*. 14, e0008962 (2020).
Chan, E. R. *et al*. Whole genome sequencing of field isolates provides robust characterization of genetic diversity in *Plasmodium vivax*. *PLoS Negl. Trop. Dis*. 9, e1811 (2012)
Chen, S. B. *et al*. Whole-genome sequencing of a *Plasmodium vivax* clinical isolate exhibits geographical characteristics and high genetic variation in China-Myanmar border area. *BMC Genomics*. 18(1):131 (2017)
Daron, J. *et al.* Population genomic evidence of *Plasmodium vivax* Southeast Asian origin. Sci Adv 7, (2021).
Delgaddo-Ratto, C. *et al*. Population Genetics of *Plasmodium vivax* in the Peruvian Amazon. *PLoS Negl. Trop. Dis*. e0004376 (2016)
Ibrahim, A. *et al*. Population-based genomic study of *Plasmodium vivax* malaria in seven Brazilian states and across South America. Lancet Reg Health Am 18, 100420 (2023).
Popovici, J. *et al*. Genomic Analyses Reveal the Common Occurrence and Complexity of *Plasmodium vivax* Relapses in Cambodia. *mBio*. 1, e01888-17 (2018).

Variant calling and genotype filtering

Variant calling data processing was performed using the vivax Genomic Epidemiology Network (vivaxGEN) Next Generation Sequencing (NGS) variant calling pipeline (https://github.com/vivaxgen/ngs-pipeline). The vivaxGEN NGS pipeline employs the GATK4 best-practice workflow[12]. For the current study, the parameters used in the MalariaGEN Pv4 variant calling pipeline were followed as closely as possible to derive genotype calls at the 911,901 high quality biallelic SNPs described in the Pv4 dataset[3]. In brief, the reads of each sample (827 of external source and 1,895 from Pv4) were mapped to both the PvP01 *P. vivax* and human reference sequences using *bwa-mem2* version 2.2.1 (https://github.com/bwa-mem2/bwa-mem2) to yield BAM files. The BAM files were then filtered to only keep correctly paired reads mapped to *P. vivax* for further processing. Read deduplication was then performed using *samtools markdup*. For samples with multiple SRA accession numbers, the deduplicated BAM files from each SRA were then merged using *samtools merge* to obtain a single BAM file per sample. Base calibration was performed using *GATK4.3 BaseRecalibrator/ApplyBQSR* with 1,303,984 known variants from the Pv4 dataset (variants annotated as PASS in the open-access Pv4 VCF file; https://www.malariagen.net/data_package/open-dataset-plasmodium-vivax-v4-0/) as the calibrator to obtain

a calibrated BAM file of each sample. *GATK4.3 HaplotypeCaller* was used to perform variant calling on the calibrated BAM files to yield GVCF files, and finally joint variant calling utilizing *GATK4.3 GenotypeGVCFs* was conducted on those GVCF files to obtain raw merged VCF data.

The raw VCF data was annotated for minimum depth of 5 to successfully call a genotype (<5 reads defined as a genotype failure/missing genotype), and a minimum depth of 2 reference and 2 alternate alleles to define a genotype as heterozygote; these annotations were conducted using an in-house Python script contained within the vivaxGEN NGS pipeline. The annotated VCF data were then filtered for the 1,303,984 known variants in Pv4 set using *bcftools view* command.

Neighbor-joining tree construction and F_{WS} calculation

The 827 non-Pv4 samples with <50% genotype failures across the selected microhaplotype SNPs (494 biallelic SNPs within the exemplar 100-microhaplotype high diversity panel) were selected for neighbor-joining analysis, resulting in 324 samples from 19 countries as outlined below:

Country	Number samples	Number samples in microhaplotype evaluation	Region*
Afghanistan	22	22	WAS
Bangladesh	1	1	WSEA
Brazil	87	87	SAM
Cambodia	56	56	ESEA
China	6	6	ESEA
Colombia	4	4	SAM
Eritrea	12	12	AF
Ethiopia	8	8	AF
Guyana	3	3	SAM
India	33	33	WAS
Madagascar	3	3	AF
Mauritania	10	10	AF
Pakistan	32	32	WAS
Panama	33	0	SAM
Papua New Guinea	1	1	OCE
Philippines	1	1	MSEA
Salvador	1	1	SAM
Sudan	8	8	AF
Uganda	3	3	AF

*AF=Africa, ESEA=East Southeast Asia, MSEA=Maritime Southeast Asia, OCE=Oceania, SAM=South America, WAS=West Asia and WSEA=West Southeast Asia.

The 324 filtered samples in the independent validation dataset were combined with 1,031 Pv4 high-quality samples (samples that were annotated as Analysis_set in the Pv4 metadata; https://www.malariagen.net/data_package/open-dataset-plasmodium-vivax-v4-0/). The combined set was then filtered for variants with sample missingness <0.05 (5%), filtered for variants with minor allele count (MAC)=2, filtered for samples with variant missingness <0.5 (50%), and re-filtered for variants with MAC=2 again, consecutively. The final filtered dataset comprised 329,371 biallelic variants and 1,354 samples (of which 324

were independent validation samples and 1,030 Pv4 samples). All filtering was performed using *bcftools view* command.

A distance matrix based on major allele proportional genetic distance was calculated from the filtered dataset (n=1,354 samples), and a neighbor-joining tree was generated and plotted using the R software *ape* package[13]. The branches in the neighbour-joining tree were then coloured according to (i) country of origin for both Pv4 and independent validation samples, (ii) country of origin for independent validation samples only, and (ii) regional classification as per the Pv4 genetic clustering patterns[3]. The F_{WS} metric, which provides a measure of within-host inbreeding that can be used to gauge whether an infection is likely polyclonal or monoclonal, was calculated on the same dataset using the R software *moimix* package (https://github.com/bahlolab/moimix). As per the Pv4 polyclonal classifications[3,14], an F_{WS} threshold of <0.95 was used to classify infections as likely being polyclonal.

Identity-by-descent estimation

Correlation between microhaplotype-based identity-by-descent (IBD) and whole genome-based IBD was calculated on the filtered independent validation samples belonging to the four regional groups (AF, ESEA, SAM and WAS) that comprised >30 samples, for a total of 288 samples as detailed below.

Region	Number of Samples
AF	44
ESEA	62
SAM*	95
WAS	87

*Note that samples from Panama were excluded from the SAM region owing to a clonal expansion that could bias the IBD evaluation[4].

Using the 494 biallelic SNPs within the 100-microhaplotype high diversity panel, IBD was estimated using *hmmIBD* version 3[15], with additional parameters min_snp_sep =0 and max_all=100. Genome-wide estimates of IBD were conducted using *hmmIBD* with the default values. The correlation between the microhaplotype and genome-wide IBD estimations was illustrated with scatter plots comprising colour-coding by pairwise-missing data/genotype failures at the 494 microhaplotype-derived SNPs (i.e. missingness when one or both samples in a pair had a genotype failure/missing call). Scatter plots were generated using an in-house Python script. The magnitude and significance of the correlations was assessed using Spearman's rho statistic (using a paired test).

References

1 Battle, K. E. *et al.* Mapping the global endemicity and clinical burden of Plasmodium vivax, 2000-17: a spatial and temporal modelling study. *Lancet* **394**, 332-343, doi:10.1016/S0140-6736(19)31096-7 (2019).

2 Trimarsanto, H. *et al.* A molecular barcode and web-based data analysis tool to identify imported Plasmodium vivax malaria. *Commun Biol* **5**, 1411, doi:10.1038/s42003-022-04352-2 (2022).

3 MalariaGen *et al.* An open dataset of Plasmodium vivax genome variation in 1,895 worldwide samples. *Wellcome open research* **7**, 136, doi:10.12688/wellcomeopenres.17795.1 (2022).

4 Buyon, L. E. *et al.* Population genomics of Plasmodium vivax in Panama to assess the risk of case importation on malaria elimination. *PLoS neglected tropical diseases* **14**, e0008962, doi:10.1371/journal.pntd.0008962 (2020).

5 Benavente, E. D. *et al.* Distinctive genetic structure and selection patterns in Plasmodium vivax from South Asia and East Africa. *Nature communications* **12**, 3160, doi:10.1038/s41467-021-23422-3 (2021).

6 Ibrahim, A. *et al.* Population-based genomic study of Plasmodium vivax malaria in seven Brazilian states and across South America. *Lancet Reg Health Am* **18**, 100420, doi:10.1016/j.lana.2022.100420 (2023).
7 Daron, J. *et al.* Population genomic evidence of Plasmodium vivax Southeast Asian origin. *Sci Adv* **7**, doi:10.1126/sciadv.abc3713 (2021).
8 Chan, E. R. *et al.* Whole genome sequencing of field isolates provides robust characterization of genetic diversity in Plasmodium vivax. *PLoS neglected tropical diseases* **6**, e1811, doi:10.1371/journal.pntd.0001811 (2012).
9 Chen, S. B. *et al.* Whole-genome sequencing of a Plasmodium vivax clinical isolate exhibits geographical characteristics and high genetic variation in China-Myanmar border area. *BMC genomics* **18**, 131, doi:10.1186/s12864-017-3523-y (2017).
10 Delgado-Ratto, C. *et al.* Population Genetics of Plasmodium vivax in the Peruvian Amazon. *PLoS neglected tropical diseases* **10**, e0004376, doi:10.1371/journal.pntd.0004376 (2016).
11 Popovici, J. *et al.* Genomic Analyses Reveal the Common Occurrence and Complexity of Plasmodium vivax Relapses in Cambodia. *mBio* **9**, doi:10.1128/mBio.01888-17 (2018).
12 McKenna, A. *et al.* The Genome Analysis Toolkit: a MapReduce framework for analyzing next-generation DNA sequencing data. *Genome research* **20**, 1297-1303, doi:10.1101/gr.107524.110 (2010).
13 Paradis, E., Claude, J. & Strimmer, K. APE: Analyses of Phylogenetics and Evolution in R language. *Bioinformatics* **20**, 289-290, doi:10.1093/bioinformatics/btg412 (2004).
14 Auburn, S. *et al.* Characterization of within-host Plasmodium falciparum diversity using next-generation sequence data. *PloS one* **7**, e32891, doi:10.1371/journal.pone.0032891 (2012).
15 Schaffner, S. F., Taylor, A. R., Wong, W., Wirth, D. F. & Neafsey, D. E. hmmIBD: software to infer pairwise identity by descent between haploid genotypes. *Malaria journal* **17**, 196, doi:10.1186/s12936-018-2349-7 (2018).
