# Supplementary material for: Lineage-informative microhaplotypes for recurrence classification and spatio-temporal surveillance of Plasmodium vivax malaria parasites
Source: Nat Commun. 2024 Aug 8;15:6757. doi: 10.1038/s41467-024-51015-3 (PMC11310204; doi:10.1038/s41467-024-51015-3)
Supplement: Supplementary file 3 — Description of Additional Supplementary Information [file 41467_2024_51015_MOESM3_ESM.docx]

**Description of additional supplementary files**

Title: Supplementary Data 1

Description: High-quality monoclonal sample information.

Title: Supplementary Data 2

Description: Microhaplotype panel marker selection information.
